# Changes in Transcranial Sonographic Measurement of the Optic Nerve Sheath Diameter in Non-concussed Collegiate Soccer Players Across a Single Season

**DOI:** 10.7759/cureus.3090

**Published:** 2018-08-03

**Authors:** Saeed S Sadrameli, Marcus S Wong, Rasadul Kabir, Jonathan R Wiese, Kenneth Podell, John J Volpi, Rajan R Gadhia

**Affiliations:** 1 Neurosurgery, Houston Methodist Neurological Institute, Houston, USA; 2 Radiology, Houston Methodist Neurological Institute, Houston, USA; 3 Neurology, Houston Methodist Neurological Institute, Houston, USA

**Keywords:** optic nerve sheath diameter, sports concussion, women's soccer

## Abstract

Introduction

Bedside ultrasound measurement of the optic nerve sheath diameter (ONSD) is emerging as a non-invasive technique to evaluate and predict raised intracranial pressure (ICP) in both children and adults. The prognostic value of increased ONSD on brain computed tomography (CT) scan has previously been correlated with increased intensive care unit (ICU) mortality in patients with severe traumatic brain injury (TBI). Previous studies have also evaluated the association between high-contact sports, such as soccer, and TBI; however, the related changes in ONSD are still unknown. The aim of this study was to evaluate for the natural evolution of changes in ONSD in athletes who participate in high-contact sports.

Methods

In this prospective observational study, volunteers from a collegiate women’s soccer team underwent the measurement of ONSD with transcranial Doppler (TCD). ONSDs were measured during the initial visit during the pre-season period and again at the three-month follow-up. A single experienced neuro-sonographer performed all measurements to eliminate any operator bias.

Results

Twenty-four female college soccer players between the ages of 18 and 23 were included in this analysis. Mean ONSD during the initial pre-season clinic visit and the three-month follow-up were 4.14±0.6 mm and 5.02±0.72 mm, respectively (P < 0.0001). A two-tailed t-test analysis was performed, which resulted in a t-value of 4.76 and P < 0.00001. The average ONSD measured during the post-season follow-up showed a 21.3% increase compared to the baseline.

Conclusion

The evaluation of high-contact sports athletes is limited due to the lack of objective radiologic and diagnostic tools. Moreover, in an athlete suffering a concussion, return-to-play decisions are heavily dependent on the symptoms reported by the athletes. In our analysis of collegiate women’s soccer players, active participation in soccer competitions and practice may be associated with an increase in ONSD, independent of concussions. Further studies are underway to evaluate the clinical significance of these findings as well as possible correlations between concussions and changes in ONSD.

## Introduction

Seventy-five percent of traumatic brain injury (TBI) cases are classified as mild, with an annual incidence of 300-500/100,000, imposing an estimated annual financial burden of $17 billion [1-2]. Like many contact sports, soccer is not immune to such injuries. Soccer is currently the most popular and fastest-growing sport worldwide, with over 265 million active players and 27 million playing the sport within Canada and the US alone [3]. According to a study published by Zuckerman et al. in 2015, annual national estimates of reported sport-related concussion (SRC) in the National Collegiate Athletic Association (NCAA) between 2009 and 2014 indicate that women’s soccer has the second-largest incidence of SRC and is ranked fourth among all sports responsible for recurrent concussions in student-athletes. The most common mechanisms of concussion in soccer involve player or ball contact while heading the ball and player contact during general play [4]. In this study, a comparison of incidents of reported concussions in men’s and women’s soccer indicated higher concussion rates overall (RR = 1.83; 95% CI, 1.34-2.51) and in competitions (RR = 2.00; 95% CI, 1.35-2.96) in female athletes [3].

Concussion is defined as an alteration of mental status as a result of a blow to the head or body [5]. While the prevention and treatment of concussion in the general population have been studied extensively, the management of concussion in athletes deserves more investigation, mainly to address one question: “when is it safe for the athlete to return to play?” [6]. Unfortunately, due to a lack of readily available diagnostic tests, such as serum biomarkers or a portable imaging device, it is challenging to make an unbiased decision regarding athletes’ return to play at the sidelines. The Sport Concussion Assessment Tool (SCAT) currently provides the most objective data to evaluate the readiness and safety of athletes prior to return to sport. While managing patients according to their clinical symptoms is acceptable, it does not account for the cases of underreporting in order to accelerate a return to play or overemphasis due to fear or secondary gain. While the former poses more risk and can increase the risk of second impact syndrome or more severe subsequent concussions, both phenomena cloud accurate clinical judgment [7-8]. Currently, athletes diagnosed with concussion undergo multiple post-concussive assessment tools, including graded symptom checklists, standardized assessment of concussion, neuropsychological assessments, and SCAT in order for the athletic trainer to diagnose at the sidelines [5]. While these assessment tools allow for clinical judgment with reasonable accuracy, they are not the most ideal tools to be utilized at the sidelines due to the length and extensive nature of the questionnaire.

In the past decade, multiple studies have focused their effort on analyzing biomarkers released in serum or cerebrospinal fluid (CSF) as a result of concussion-induced neuronal or glial injury. Measuring and trending such biomarkers allow for an objective tool to analyze the severity of the injury and the possible timeline for recovery. There are, however, challenges associated with a biomarker analysis, such as their specificity for neuronal injury and the complexity of assays needed to detect these markers [9-10]. More recently, the role of non-invasive monitoring for the measurement of intracranial pressure and cerebral blood flow has gained popularity. The results of pilot studies in the past implied changes in intracranial pressure and cerebral autoregulation following a concussion [11-12].

The purpose of this study was to propose a novel objective method to evaluate possible fluctuations in patients’ intracranial pressure in a high-contact sport.

## Materials and methods

After appropriate institutional review board (IRB) approval was obtained, a prospective single-center study within the subject pre-post season design was conducted to study the optic nerve sheath diameters (ONSDs) in a collegiate women’s soccer team. Appropriate informed consents were obtained. Between July and December of 2012, healthy female volunteers from division I collegiate female soccer team without a prior diagnosis of psychiatric problems or concussion underwent two measurements of ONSD: first, during the pre-season period and, again, at post-season follow-up, within three weeks of end of season. None of the subjects in this data analysis were reported or diagnosed with concussion. All measurements were performed by a single, experienced, certified neuro-sonographer using the same ultrasound machine to eliminate any operator bias.

**Figure 1:**
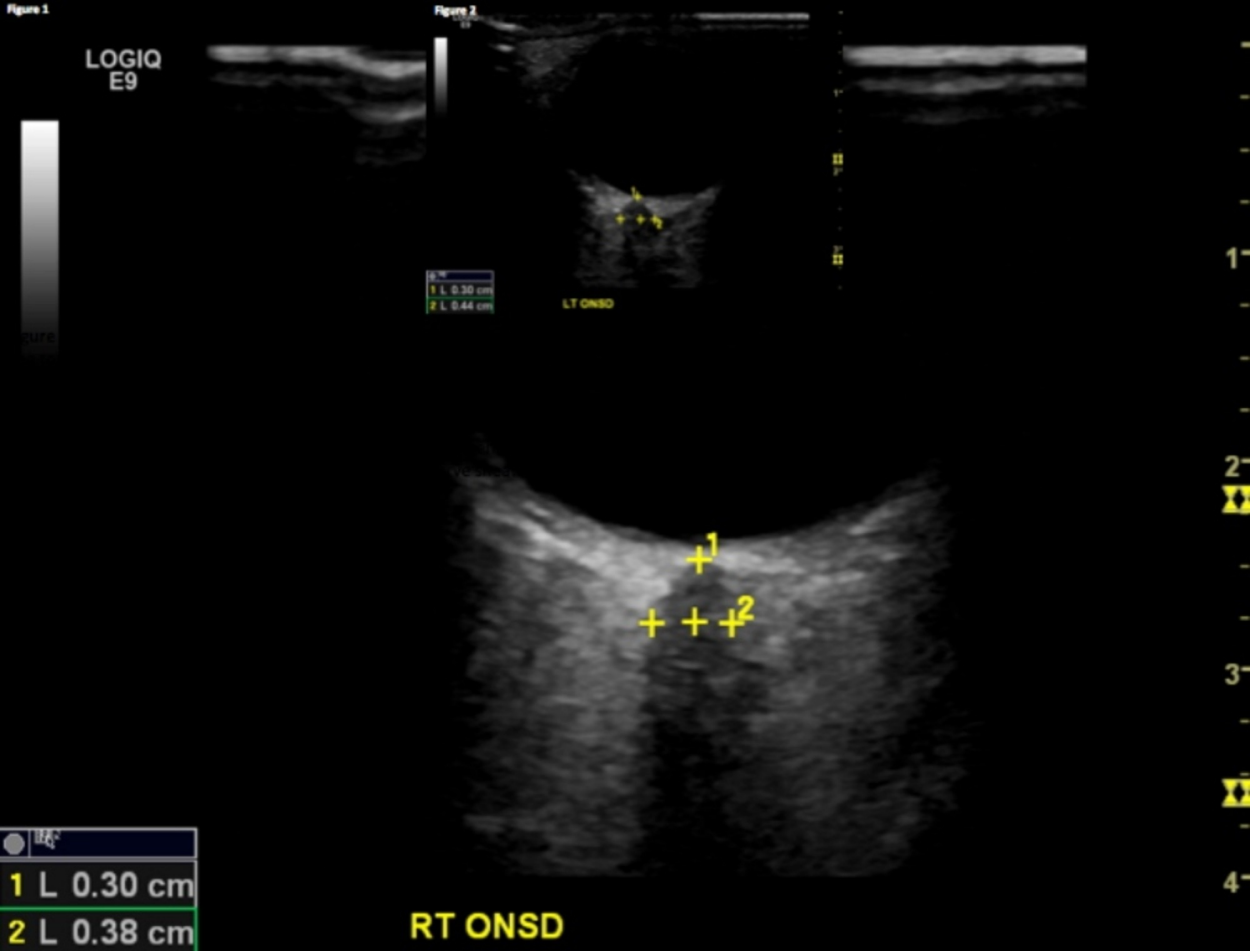
Pre-season measurement. The top value represents the distance from the optic disc/globe to the optic nerve sheath (normal = 0.29 to 0.3 cm). The bottom number indicates the normal ONSD (normal < 0.4 cm).

**Figure 2:**
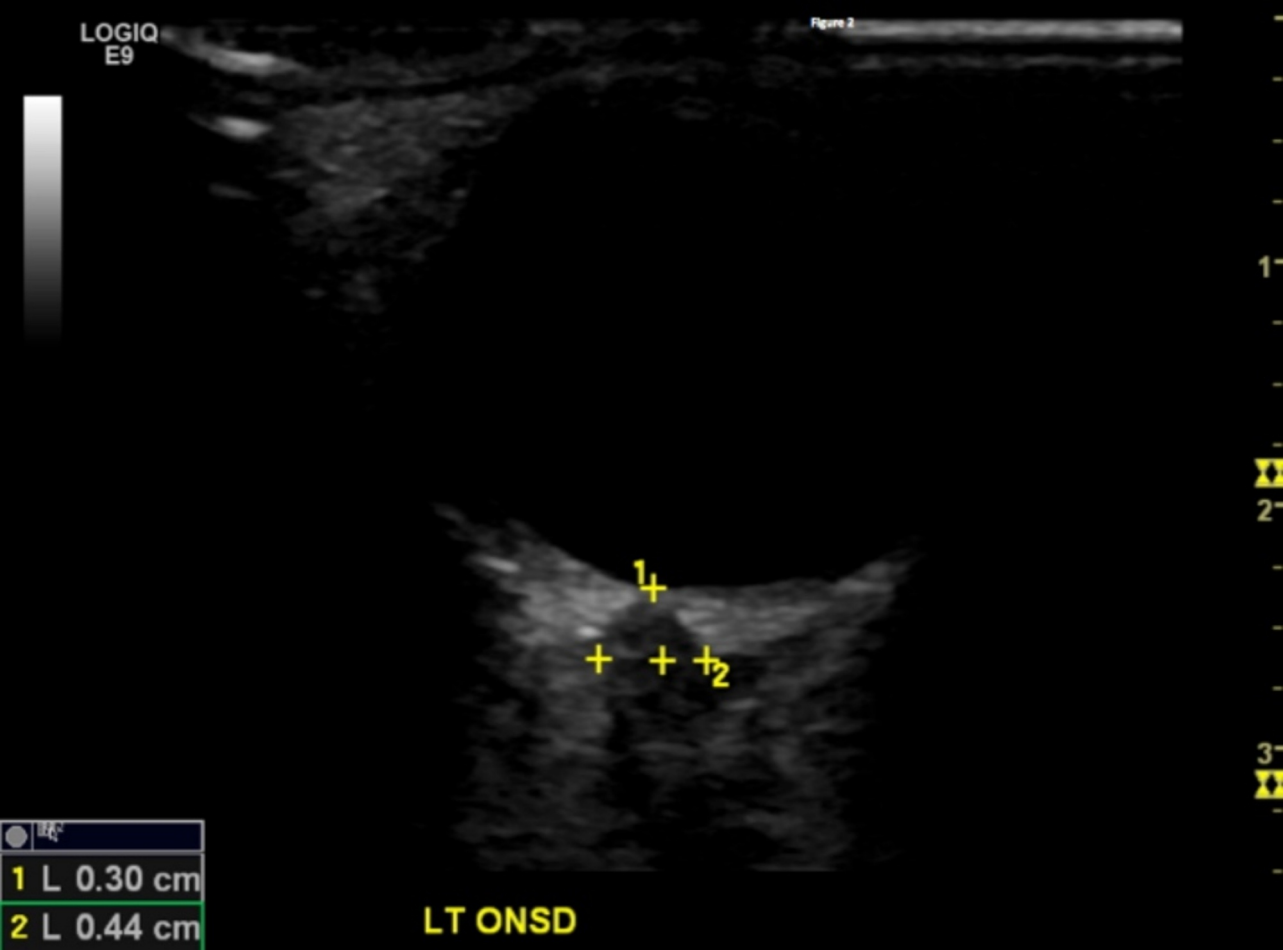
Post-season measurement. The top value represents the distance from the optic disc/globe to the optic nerve sheath (normal = 0.29 to 0.3 cm). The bottom number represents the enlarged ONSD (normal < 0.4 cm).

A high-frequency General Electric (Fairfield, CT, US) LOGIQ E9 9 MHz linear probe was utilized for this study. The probe was placed over the closed eyelid with the patient in supine position. A small amount of ultrasound gel was used to avoid any discomfort for the patient. The probe was held in a transverse plane with the beam directed posteriorly towards the optic disc complex. The patient was asked to look down while keeping their eyes closed. The ONSD was measured 3 mm behind the globe using an electronic caliper. Each diameter was measured three times and the mean value was used for the final analysis (Figures 1-2).

## Results

**Figure 3:**
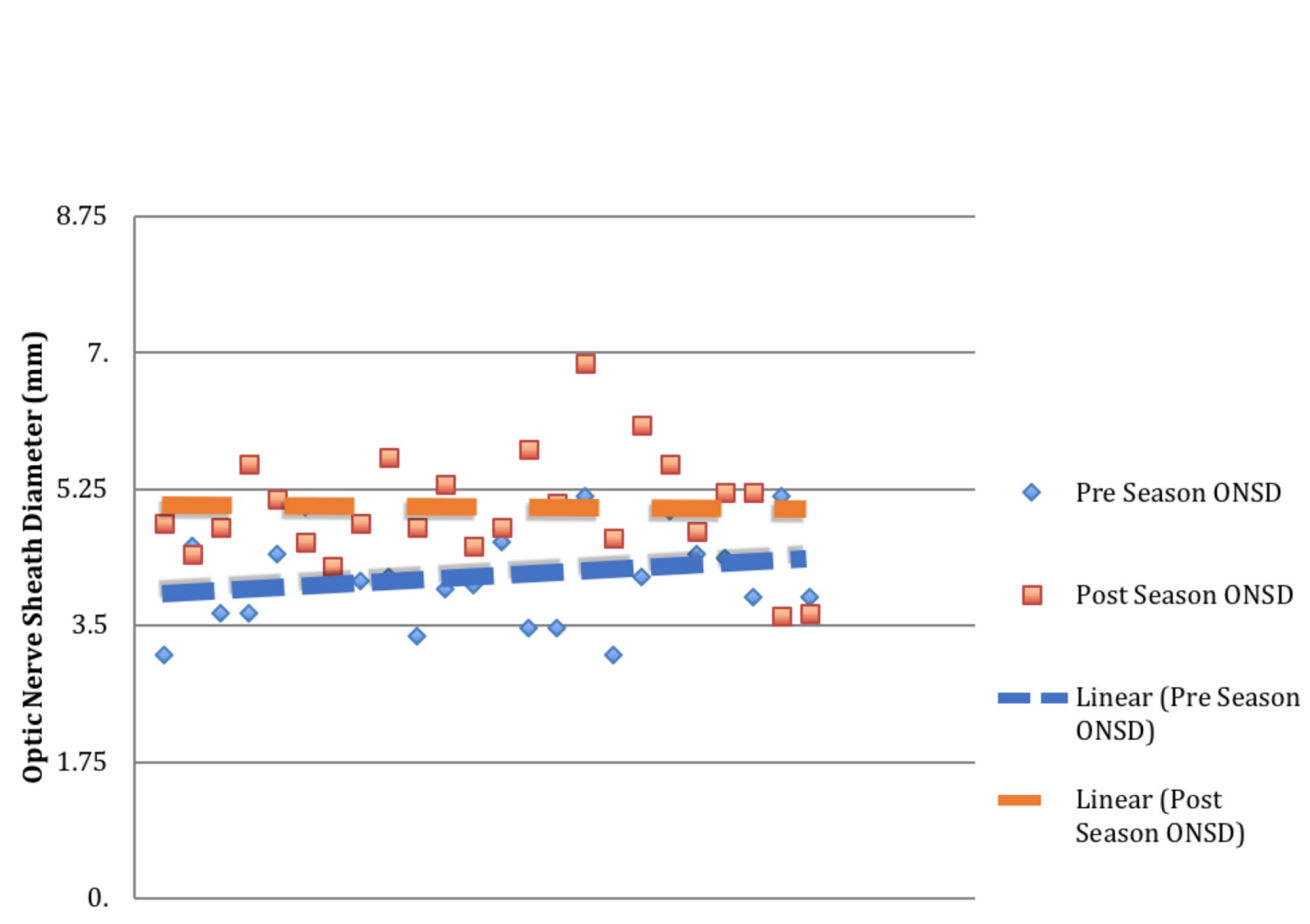
Pre-season and post-season optic nerve sheath diameters.

Twenty-four female college soccer players between the ages of 18 and 23 were included in this analysis. Mean ONSD during the initial pre-season clinic visit and the post-season follow-up were 4.14±0.6 mm and 5.02±0.72 mm, respectively (P < 0.0001, t = 4.69, Cohens D = 1.33). The average ONSD measured at the end of the season showed a 21.3% increase compared to the baseline (Figure 3).

## Discussion

Sport-related concussion (SRC) has been a topic of interest in the past two decades with 3.8 million instances reported by the Centers for Disease Control (CDC) in any given year [13]. The implications of concussion have been of recent interest in the setting of professional- and non-professional-level sports. Conventional imaging studies are typically performed hours after concussions at the earliest and lack sensitivity for any clinical utility for on-field use. With recent technological evolution, a portable imaging device can be utilized on the sidelines to provide specific and sensitive data and aid in the rapid diagnosis of concussion seconds after impact.

Sonography, as an example, can be used not only for structural visualization but also for dynamic and functional monitoring. TCD, which can be utilized with ease and without any known harm to patients, has been a large focus of evaluations. The optic nerve is surrounded by cerebrospinal fluid, and thus measurement of ONSD by transcranial ultrasound has been studied as an indirect marker of elevated intracranial pressure (ICP). In 2013, Legrand et al. utilized the head computed tomography (CT) of 100 severe TBI patients to measure ONSD [14]. Since then, multiple studies have investigated the role of sonographic ONSD measurements in the prediction of ICP [6,15-17]. Assuming that some degree of elevated ICP takes place after mild TBI, measuring ICP non-invasively could potentially offer a sensitive and specific tool to prognostication and return-to-play guidelines.

This study sought to evaluate the changes in ONSD that take place in contact-sport athletes across the season. To our knowledge, it is the first to study ONSD prospectively in soccer athletes at a higher risk for head trauma. Our findings revealed a significant 21.3% increase in ONSD in the post-season compared to the baseline measurements. This suggests to us that even without a clinical diagnosis of concussion, there are changes that take place consistent with mild, asymptomatic elevations in ICP. One could attribute changes in diameter to elevations in blood pressure that led to increases in cerebral blood flow and/or volume and, ultimately, the intracranial pressure. Another hypothesis is a compilation of recurrent minor head traumas that are inherent to the nature of the sport. Due to the small size of our cohort, it would be interesting to study a larger population of athletes and perform measurements post-concussion at multiple points in time to look for pseudo-normalization, knowing what we know from our current study. The limitations of the current study are as follows: first, there is no normal control group for a comparison of ONSD. This limits our understanding of data collected, specifically when comparing pre- and post-season ONSD values. While we assume the pre-season values are closer to the average normal optic nerve sheath diameters presented in literature (< 4.5 mm), we do not have a statistical comparison. Second, we did not account for certain variables, such as blood pressure, pulse, or number of headers, throughout the season as possible contributors to the changes seen in pre- and post-season ONSD measurements. Finally, factors such as blood volume and dehydration should be considered as possible confounders since pre-season measurements are typically performed in the summer and post-season visits are at the end of fall when the temperature is much cooler. Future studies with a control group are needed to account for such variables and to provide a more thorough and unbiased analysis.

## Conclusions

This study sought to evaluate changes in ONSD that take place in collegiate, female soccer athletes throughout the season and found a significant 21.3% average increase in ONSD in the post-season period, independent of concussions. The results of the current study can perhaps be used to explain the natural history of ONSD in athletes prone to head injury, such as soccer players. From our data alone, it is difficult to make any definitive conclusions about ICP and ONSD in the setting of collegiate sports (soccer in particular), but we show a trend that warrants further study. Ultimately, sonographic evaluation of ONSD on the sidelines could lead to a more accurate and timely diagnosis of concussion, which, in turn, would prevent further injuries such as second-impact syndrome.
